# Neuropsychophysiological Measures of Alcohol Dependence: Can We Use EEG in the Clinical Assessment?

**DOI:** 10.3389/fpsyt.2020.00676

**Published:** 2020-07-17

**Authors:** Rosa Jurado-Barba, Ana Sion, Andrés Martínez-Maldonado, Isabel Domínguez-Centeno, Julio Prieto-Montalvo, Francisco Navarrete, María Salud García-Gutierrez, Jorge Manzanares, Gabriel Rubio

**Affiliations:** ^1^ Biomedical Research Institute, Hospital 12 de Octubre, Madrid, Spain; ^2^ Department of Psychology, Education and Health Science Faculty, Camilo José Cela University, Madrid, Spain; ^3^ Addictive Disorders Network, Carlos III Institute, Madrid, Spain; ^4^ Department of Clinical Neurophysiology, Hospital Gregorio Marañón, Madrid, Spain; ^5^ Neuroscience Institute, Miguel Hernández University-CSIC, Alicante, Spain; ^6^ Medicine Faculty, Complutense de Madrid University, Madrid, Spain

**Keywords:** alcohol dependence, electroencephalogram, endophenotypes, incentive salience, negative emotionality, executive dimension, event-related potential

## Abstract

Addiction management is complex, and it requires a bio-psycho-social perspective, that ought to consider the multiple etiological and developmental factors. Because of this, a large amount of resources has been allocated to assess the vulnerability to dependence, i.e., to identify the processes underlying the transition from substance use to dependence, as well as its course, in order to determine the key points in its prevention, treatment, and recovery. Consequently, knowledge \from neuroscience must be taken into account, which is why different initiatives have emerged with this objective, such as the “Research Domain Criteria” (RDoC), and the “Addiction Neuroclinical Assessment” (ANA). Particularly, neuropsychophysiological measures could be used as markers of cognitive and behavioral attributes or traits in alcohol dependence, and even trace clinical change. In this way, the aim of this narrative review is to provide an overview following ANA clinical framework, to the most robust findings in neuropsychophysiological changes in alcohol dependence, that underlie the main cognitive domains implicated in addiction: incentive salience, negative emotionality, and executive functioning. The most consistent results have been found in event-related potential (ERP) analysis, especially in the P3 component, that could show a wide clinical utility, mainly for the executive functions. The review also shows the usefulness of other components, implicated in affective and substance-related processing (P1, N1, or the late positive potential LPP), as well as event-related oscillations, such as theta power, with a possible use as vulnerability or clinical marker in alcohol dependence. Finally, new tools emerging from psychophysiology research, based on functional connectivity or brain graph analysis could help toward a better understanding of altered circuits in alcohol dependence, as well as communication efficiency and effort during mental operations. This review concludes with an examination of these tools as possible markers in the clinical field and discusses methodological differences, the need for more replicability studies and incipient lines of work. It also uses consistent findings in psychophysiology to draw possible treatment targets and cognitive profiles in alcohol dependence.

## Introduction

Addictions continue to be a public health problem, despite the continued efforts by different types of professionals. That is why great efforts are being made from a preventive and therapeutic point of view, although it seems that these efforts are not being effective. Both preventive and therapeutic strategies include information from a psychosocial approach, and more recently from the neurosciences, not only from the field of psychopharmacology but also genetics and neuroimaging (1).

However, the results of these procedures, although highly evidenced and confirmed, are quite heterogeneous, possibly in relation to the clinical and methodological characteristics of the studies available in this field (1). For instance, different types of patients, type of consumption, withdrawal periods, and of course different type of interventions. In fact, treatment does not bring with certainty the abstinence maintenance and a great number of patients go through relapse in the first follow-up year (2–4).

To cope with this complexity, there is a consensus that in order to improve the outcomes of behavioral interventions in alcohol use disorders (AUD) it is necessary to understand the mechanisms underlying the behavioral change in effective treatments. From this standpoint, building a strong foundation for alcohol dependence treatment includes answering the question of why and how, not just whether, a treatment is effective (5).

Research on the mechanisms of effective AUD treatments that underlie behavior change has been focusing on cognitive neuroscience. Dysfunctional processes that maintain AUD, such as craving, withdrawal, lapse, and relapse are understood by studying the functioning of cognitive processes, such as attention or motivation, and the underlying neural systems.

The Research Domain Criteria (RDoC) is a framework that allows the study of psychiatric disorders from this point of view since its objective is the analysis of what may cause the symptoms, rather than the symptoms themselves. It even allows the search and validation of biomarkers (6). It takes into consideration five dimensions for all possible pathologies: negative valence systems, positive valence systems, cognitive systems, social systems, and activation supervising systems (7). Therefore, patients could be evaluated in each one of these dimensions at different levels of analysis, from basic disciplines involving genetics, molecules, cells or neuroanatomical circuits, including physiology to clinical or behavioral measures, such as neuropsychological assessment, self-informed measures or behavioral paradigms (8, 9).

This approach makes it possible to characterize the psychological attributes by being analyzed in cognitive, social, emotional, and behavioral terms, with measures coming from basic science and clinical area (10).

Following this framework, the Addiction Neuroclinical Assessment (ANA) considers the same levels of analysis, selecting three domains as essential; these are the incentive salience, negative emotionality, and executive abilities (8). Therefore, each level of analysis within the dimensions proposed by ANA could be considered as a follow-up measure of the course of substance dependence, perhaps understanding it as an intermediate endophenotype. The disorder is understood as an active process, not an endpoint, and each of the measures obtained at different levels of analysis would be considered a marker of this process (7, 11). In this way, in this study we summarize the relationship between the pathological consumption of alcohol and the use of EEG as a measure of cognitive components assessment, according to ANA dimensions.

Besides the changes produced by moderate alcohol consumption in the brain electrical activity (12–14), in alcohol dependence EEG measures have been employed as markers of structural and functional changes that rise as a consequence of continuous and pathological consumption. Hence, there is considerable bibliography available with respect to these changes (15–17). By using the brain electrical activity, we can obtain different types of information, useful for understanding alcohol dependence processes; the most frequent one in the scientific literature is the one provided by event related potentials, in the sensorial, cognitive or motor modality. They are represented by the average electrical activity appearing after each event, and are composed by different components, which are named according to their polarity (positive or negative) and the moment when they appear (in milliseconds).For instance, the P300 component would reflect a positive deflection approximately 300 ms after stimulus presentation (16). A different type of analysis is the one brought by brain oscillations, their pattern would reflect the sum of postsynaptic potentials generated by a neuronal field close to an electrode. In this way, the number of oscillations (measured in Hz) determines the traditionally known frequency bands (e.g., delta, theta, alpha, beta, and gamma bands). All of them have been explored in alcohol dependent patients, however, the most frequent findings point to beta rhythms (12–28 Hz) alterations, observed even during the abstinence period and their offspring (15). According to these basic concepts different, conditions of EEG recordings arise (e.g., resting-state or event-related activity), as well as different methods of analysis, which are addressed later in this work, such as the synchrony in brain rhythms between different locations.

The measures obtained by means of neuropsychophysiological assessment are an example of possible biomarkers that reflect, in an indirect but objective way, the cognitive processes that give rise to the problematic behavior of alcohol consumption. Several benefits could be drawn from these type of measures: 1) It allows the reflection of the related brain activity, with a great temporal, “instantaneous and continuous” precision, in words of Campanella (18); 2) When the employed paradigm is well-characterized from the cognitive and methodological points of view, the neural pattern can reflect the psychological features that contribute to the disorder, even when no observation was made from the psychopathological point of view (18, 19), 3) In addition, it is a measure that escapes the patient’s voluntary control, for example, it reflects the lack of inhibitory control or craving, even when the patient is not aware of it (20).

The suggestion that neuropsychophysiological measures, namely, event-related potentials (ERP) could be biomarkers, e.g., the P300 component, or other intermediate endophenotypes, such as N170 or N200, is a well-established line of work, from the research standpoint. Nonetheless, these are not consistently used in clinical evaluation. The aim of this revision is to provide an overview, through a narrative revision of the literature, of the most solid results in the different neuropsychophysiological measures that can be obtained by using electroencephalographic (EEG) measures.

## Dimensions of Addiction Neuroclinical Assessment and Neuropsychophysiological Evaluation

### Incentive Salience

Incentive salience is one of the main objectives of evaluation inside ANA frameworks. As defined by early theories of addiction and I-RISA model (21), incentive salience can be understood as the narrowing of the attentional focus on the substance and the related contextual cues, that gain in motivational value and appetitive properties, in detriment of natural reinforcers, changing the entire reward system. A sensitization to the substance takes place, based on associative and conditioned reinforcement mechanisms, where the mere presence of related cues can drive substance-seeking behavior (22, 23), by changing activation and craving states (24, 25). In alcohol dependence, alterations in the affective information processing and preferential attention toward substance cues have been described (26, 27) as well as attentional bias and interference control effects (28–30). Moreover, cue exposure is related to a higher desire and urgency consumption and increased expectancies regarding alcohol effects, as well as dependence severity (26, 27). Changes in the reward system are also observed, resulting in difficulties regarding the delay of gratification and poor loss and gain evaluation (20, 31–34). Thereby, the incentive salience construct, together with cognitive and motivational processes involved seem essential in the search for clinical markers of change through the dependence process and as vulnerability factors involved in maladaptive behaviors. These can be measured through a great variety of tools, including self-informed measures, neuropsychological tests, and behavioral paradigms, sometimes combined with neuroimaging and psychophysiological techniques. ANA model proposes specific materials for evaluating the incentive salience domain, including behavioral paradigms such as the dot-probe attentional bias task (29, 35, 36), cue-reactivity task (37) or the monetary incentive delay task (38).

Nonetheless, initiatives like ANA or RDoC encourage the use of neuroimaging tools, like psychophysiological measures. Taking into consideration that the motivational and attentional allocation changes toward substance-related cues are produced in parallel with structural and functional changes of reward and salience circuitry of the brain, the neural underpinnings of these processes become relevant in the discovery for new markers of clinical changes and vulnerability related to the dependence process. In this way, neuropsychophysiological measures enable the assessment of early stimuli evaluation (perceptive and preattentional operations), together with distractibility and the attentional biased produced by substance-related stimuli, as well as mental operations during reward processing and delay of gratification.

Early visual components, such as P1, N1, or P2, as well as the late positive potential (LPP) are ERP components usually related to early stimulus evaluation and attentional processes that can be found altered in AUD in cue reactivity tasks or attentional bias paradigms. One of the most frequent ways to evaluate the influence of alcohol cues on neuropsychophysiological activity is by using cue reactivity tasks that usually consist in the presentation of visual substance-related stimuli (images or words), and even olfactory or gustatory cues. These can be passively visualized (e.g., a bottle of whiskey or neutral content stimuli, such as a book or a pen) or can be part of a classification or a discrimination task while the electrical brain activity is being recorded.

There is evidence in the literature for an early preferential processing of substance-related cues in AUDs, shown by greater N1 amplitudes (39), an exogenous and automatic attention index, indicating a preferential attentional processing of alcohol cues, in line with motivated attention (40) and theories of addiction (41). P3 to alcohol cues in an oddball task is also found increased in alcohol dependence, indicating the alteration of higher-order processing such as controlled attentional allocation (42). This preferential processing of alcohol cues also appear in individuals at risk, ERP changes being observed in social drinkers (43) with heavy use (44), that show increased P1 latencies and LPP amplitudes toward alcohol images, and in individuals with a low sensitivity to alcohol effects, that have increased P3 amplitudes (45, 46). In this way, since early stages of alcohol consumption and in individuals at risk (low alcohol sensitivity), substance-related cues seem to be processed faster and with a high motivational salience, and ERP monitoring could help predict substance-related problems.

Alcohol salience can even dampen other demanding processes in course, heavy drinkers (47) and recently detoxified individuals (48) showing higher N2 amplitudes toward substance cues during inhibitory processes (during NoGo conditions). Thus, alcohol salience can be so powerful that other important executive processes could be affected, such as inhibitory control, essential for adaptive behaviors.

Regarding the attentional bias produced by substance-related cues, a common task is the dot-probe paradigm, that consists in the displaying of pairs of images (substance-related and neutral ones) appearing side by side, usually for 500 ms, prior to the presentation of a probe (a dot or an asterisk) replacing one of the pictures (on the left or right side of the screen). The subject must respond by indicating as quickly as possible, on which side the point has appeared, pressing one key for the right side and another for the left side. The attentional bias is typically calculated using the differences in reaction times when the dot is presented on the same side where alcohol-related pictures were (congruent trials) and when it is on the opposite side (incongruent trials). ERP analysis, in this case, would help to elucidate what happens with brain activity at several stages of the cue processing, that is, sensorial filtering, attention orientation and re-orientation, stimuli salience and arousal, and also more controlled attentional processes, even in absence of behavioral changes in reaction times or self-report measures. In the same way as cue tasks, visual probe paradigms display in individuals at risk (low alcohol sensitivity) a preferential early attention orientation, indicated by larger P1 amplitudes, and difficulties reorienting attention away from alcohol cues, reflected by larger negativity between 220 and 280 ms (49).

With respect to reward system changes, monetary incentive or choice tasks are a usual tool of evaluation of motivational decision making. This type of task usually consists in gambling tasks where participants take low or high risks in order to gain a prize (generally money). The subject must decide between gambling alternatives with higher rewards and losses (higher risk) or with lower rewards and losses (lower risk). These tasks allow us to evaluate not only risk-taking and the capacity of delaying gratification, but also the evaluation of the own decisions and responses through the task. Alcohol-dependent individuals (50) and youth at risk with families densely affected by alcoholism (51) seem to show lower amplitudes of the P3 component and lower activity in frontal areas (e.g., cingulate gyrus), during both gain and loss conditions of gambling tasks. There even seems to be a relation between risk-taking features and impulsivity (50). In this way, ERP analysis of risk and outcome evaluation could help in the discovery of vulnerability markers. Even recent alcohol intoxication seems to affect neuropsychophysiological activity during reward processing, reducing ERP positivity in 250 to 400 time-window (52), indicating an affectation of performance monitoring and feedback that drives the decision-making process during the task. This is also exposed in binge drinkers, that show alterations in automatic error processing (larger error-related negativity [ERN]) in a Go-NoGo task and in motivational processing (delayed error positivity Pe) in a risk task (53). This would indicate an affectation of controlled processes such as monitoring self-behavior since early stages of consumption.

Taking into consideration the importance of the incentive salience and its effects across the dependence course, its evaluation through ERP measures can bring more light upon the specific processes that underlie to appetitive changes, attentional interference produced by alcohol cues and craving, that motivationally drive the decision making. Moreover, changes in psychophysiological activity appear early in the course of continuous consumption, indicated by the early attentional and motivational capture by alcohol stimuli and by changes in the reward system in acute effects of alcohol administration and social drinkers. Some studies even find these changes in population at risk, namely individuals with low sensitivity to alcohol and individuals with families densely affected by alcohol dependence. Hence, early informational processing components, such as P1 or N2 or those involved in controlled attention, N2 and P3 could be assessed as possible vulnerability markers or as markers of cognitive efficacy change through time.

### Negative Emotionality

Within the ANAs framework, negative emotionality refers to the propensity to experience and react with negative emotions, such as sadness, anxiety, fear, and anger to environmental cues (54). In fact, there is enough evidence that shows how dependent individuals have clear difficulties within the emotional regulation process, namely a diminished emotional awareness and a reduced acknowledgment of other people’s emotions and their own, giving rise to an inadequate emotional adjusting in relation to environmental demands (55, 56). Furthermore, tolerating negative emotional states might become difficult and even bring people to act impulsively, making them to engage in behaviors that they find rewarding in the short-term without fully considering its risks, in order to diminish the experience of this negative affect. These negative reactions to environmental cues seem to be highly related to alcohol consumption. So much that different theoretical models consider that negative emotionality is present during different stages of the alcohol consumption cycle.

Among them, classical theoretical approaches (57) propose that deregulation in the reward system is characterized by the transition of the experience of pleasant sensations every time an individual consumes alcohol, to a progressive shift toward feelings of relief when he takes the substance, that is, taking alcohol with the whole purpose of avoiding the negative emotionality states experience during withdrawal stages (58). This leads to further consumption as a way to avoid this negative state, reinforcing the cycle. Additionally, neuroadaptations seem to persist even after prolonged abstinence, increasing the risk of relapse (59).

There is also broad evidence of the existence of negative emotionality prior to the development of AUD. This seems to play a key role in the engagement of problematic consumption as a self-regulation mechanism. Hagan et al. (60) found that a higher negative emotionality in children predicts future alcohol consumption, stress, and internalizing symptoms during adulthood. Additionally, there is evidence that the increase of alcohol consumption in adolescents is highly related to a reduction of the positive affect and an increased negative affect, giving rise to the usual anhedonia symptoms in this type of patients (61). Implying then, that negative emotionality can also be considered an intermediate endophenotype of alcoholism.

This negative emotionality is reflected at a biological level, hence psychophysiological changes can be assessed with EEG measurements. The use of EEG techniques in affective process evaluation can be challenging, due to its complexity. However, the correct implementation of EEG measures ensures a direct measurement of the affective processing, providing key temporal information of all stages of emotional processing, allowing then a better understanding of some of the alterations found in AUD patients. However, the number of studies of emotional alterations in AUDs with EEG measures is scarce.

ERP analysis is one of the most used EEG measures to study emotional processing with psychophysiological techniques. Among the ERPs related to emotional processing, we can mention the Early Posterior Negativity (EPN), P1, P2 and the LPP (62). The modulation of these components by affective information is reliable and systematically observed (63–65). LPP is characterized by a positive centro-parietal deflection, starting around 200 ms after the stimulus onset, and it is prolonged several milliseconds in time. It is modulated by the emotional content of stimuli, showing an increase in comparison with its activity under neutral stimuli. However, cognitive reappraisal related to positive emotional regulation seems to reduce LPP amplitude (66–68). In this type of tasks, subjects passively visualize images with high emotional content, and they are asked to classify them according to three dimensions: valence, arousal, and dominance. The emotional content of these images is usually related to positive appetitive stimuli (e.g., sex-related), negative threatening ones (e.g., aggressions) and sometimes motivationally relevant stimuli related to the substance (e.g., a beer).

Studies with paradigms of viewing of neutral and affective images found that alcohol consumption selectively reduces the processing of negative cues, specifically there is a decreased amplitude of LPP during the viewing of negative images, and this has been seen in healthy population after the intake of a small doses of alcohol (69) and in population at risk, such as binge drinkers (70). A reduced LPP amplitude would be indicating an early effect of alcohol consumption on the impact of negative-valence content in later information processing stages. This could be in line with theories regarding negative affect evaluation and processing alterations in alcohol dependence.

Prolonged alcohol consumption is also related to alterations in the recognition of emotional facial expressions. In emotion recognition tasks, the person is asked to identify emotional expressions on faces, usually without any context. When AUDs had to determine an emotional face expression they presented a distinct neuropsychophysiological response, indexed by a decreased amplitude of early (P1, N10, N170) and late (LPP) ERPs (71). This alteration persists after a prolonged abstinence (59, 72). These difficulties for recognizing emotional facial expressions are highly related to more interpersonal difficulties, probably leading to social isolation and to an increase of their negative emotionality (59).

In summary, both early processing (P1, N1, N170) and later (LPP) components can be used as potential markers when evaluating negative emotionality aspects, such as affective processing, emotional recognition, and appraisal. For instance, alcohol seems to affect LPP from the beginning of consumption and together with N1, they are affected even in prolonged abstinence, indicating a possible role for these components as possible biomarkers. Moreover, considering its modularity by re-appraisal, LPP could be measured through time, in relation to cognitive and emotional regulation therapy.

### Executive Dimension

Under the ANA framework, executive functions would be included within the executive domain, which comprises those higher-order processes mainly involved in the organization of behaviors, aimed at achieving future objectives (8, 73). Specifically, this domain is focused both on those processes of temporary organization of behavior such as attention, inhibition of response, planning, working memory and behavioral flexibility, as well as evaluation of future events (8). For an adequate measurement of the functions included within this domain, the authors of the ANA propose a series of assessment tests that can be widely used by both researchers and clinicians (8). However, in addition to the behavioral and self-reporting tests proposed by the ANA authors, they also recommend supplementing the use of these tests with other measures from neuroscience (8), but without specifying on any particular measure. This is because the evaluation of the executive dimension with behavioral and self-reporting tests exclusively may not identify aspects underlying these measures, such as inefficient brain functioning (74). This inefficient brain functioning may not manifest itself behaviorally and/or consciously in controlled contexts (e.g., attentional evaluation in clinical consultation), while in everyday contexts it does, putting at risk the maintenance of abstinence. In this case, psychophysiology can be very useful, because it can be sensitive to this type of information. Along these lines, quite interesting results have been found by combining different evaluation tests and different types of analysis of the electrophysiological signal.

One of the most studied tasks with the objective of obtaining a neuropsychophysiological marker of attentional control in alcohol addiction is the oddball paradigm. This task consists in the presentation of a series of infrequent stimuli (targets) during the presentation of stimuli in a frequent way (standards), allowing to evaluate the attentional processing from bottom-up as well as from top-down (75). The analysis of the electrophysiological activity recorded during the performance of this task that has been mostly carried out is that of ERPs (76–81). The task leads to the generation of different electrophysiological components, being P3a and P3b the most studied ones (76–81). These two electrophysiological components appear between 300 and 700 ms after the stimulus, and differ mainly in the cause that generates them and in the topographic distribution that they have. The first of these is the P3a component, whose evocation is produced by the absence of explicit instruction to attend to the infrequent stimulus, and has a frontal topographical distribution (17). This component would reflect the bottom-up attentional processing, because there is no controlled processing of the stimuli presented. The second component is the P3b, which has a parietal topographic distribution, and whose evocation is produced by the explicit instruction to attend to the infrequent stimulus (17). In this case, this component would reflect the top-down attentional processing, since, unlike the P3a component, here there is a controlled processing of the stimuli presented in the task.

A large number of studies using this oddball paradigm in alcohol-dependent people have found that both the P3a and P3b components have a reduced amplitude compared to healthy controls (42, 79, 80, 82–84). A result that is replicated in healthy children of people with alcohol dependence (78, 85). This alteration of the P3 component both in people with alcohol dependence and in their offspring would reflect an attentional deficit from both the bottom-up and top-down processes, which seems to be produced by basal brain hyperexcitability (17, 76, 86). The literature proposes that this abnormal brain functioning is produced by an alteration in the mechanisms of cortical inhibition, and not by the consumption of alcohol per se (although it also contributes to the general impairment of brain functioning), being a previous vulnerability that they present (17, 76, 86). Because of this, the amplitude reduction of this component is proposed as an electrophysiological marker for alcohol addiction development. However, in other studies comparing this component between people with alcohol addiction and healthy controls, such as those performed by Bauer et al. (87), Fein et al. (83) and Malone et al. (88), these differences are not found when other variables such as the presence of a life history of major depression are taken into account. The discrepancy of results between studies may be reflecting the effect of the absence of differentiation between people who develop alcohol addiction due to the presence of different previous vulnerabilities (e.g., genetics), and people who develop it secondarily as a consequence of the presence of a particular set of symptoms (e.g., social anxiety) (81, 88, 89).

An example of this idea is reflected by Fein et al. (83). These authors study if there are differences in the P3b component generated by the oddball paradigm between people with alcohol addiction in abstinence with and without major depression. They found that those with alcohol addiction and major depression had no difference in the amplitude of the P3b component compared to the healthy controls group. They conclude that the absence of differences is due to the fact that the development of alcohol dependence, in this case, was caused by excessive consumption of the substance with self-medication as the main objective (83). Along the same lines, Malone et al. (88) propose that this does not only occur with the presence of concurrent major depression, but that this effect is also observed in people diagnosed with major depression throughout life. These two studies seem to reflect, by way of example, that the P3 component evoked during the performance of the oddball paradigm could help to identify those people with altered attentional functioning as a consequence of a previous vulnerability, having clear repercussions in the choice of the most appropriate therapeutic line (e.g., pharmacological and neuropsychological vs. neuropsychological) (90–94).

Besides the oddball paradigm, the Go-NoGo paradigm is another task that has also been quite studied with the aim of obtaining other types of electrophysiological markers. This task has been used in different versions, varying some of its parameters, such as the type of stimuli presented [e.g., geometric figures (74), neutral stimuli or alcohol-related stimuli (95)]. However, the basic design of the task consists of presenting a series of Go stimuli more frequently to which the participant has to give some kind of response, interspersing NoGo stimuli less frequently, where the participant has to inhibit his response. Although apparently both the oddball and the Go-NoGo paradigms are very similar, the main difference is that the Go-NoGo paradigm requires greater involvement in the task by the participant, while in the oddball paradigm the necessary involvement is lower. Precisely because of this, the cognitive process that allows us to evaluate this task is the inhibitory capacity and the influence of different stimulation conditions on it.

With the use of the Go-NoGo paradigm, and as with the oddball paradigm, the analysis of ERPs has been the most carried out, which has provided the most consistent results around the N2 and P3 components (96–99). On the one hand, the negative component N2 appears around 250 ms after the presentation of the NoGo stimulus with a front-central distribution, with greater amplitude in the frontal region (98). The literature proposes that this component reflects the subject’s ability to recognize the need to inhibit the response to a stimulus of these characteristics, where a greater amplitude of the component would reflect a better recognition of this need to inhibit (98, 100). On the other hand, the positive component P3 appears between 300 and 600 ms after the presentation of the NoGo stimulus with a fronto-centro-parietal distribution, with greater amplitude in the central region (96). In this case, the literature proposes that this component reflects the subject’s ability to carry out an effective inhibition of the motor response, where a greater amplitude of the component would reflect a better inhibition of the response (101). Specifically, in people with alcohol addiction, the literature shows that both N2 and P3 can be reduced compared to healthy participants, reflecting an inhibitory deficit at one or both levels even when behavioral outcomes do not reflect this deficit (96–99).

In summary, the tasks with more clinical evidence for the evaluation of the executive dimension with electrical brain activity are the oddball and Go-NoGo paradigms. In the case of the oddball paradigm, the cognitive process evaluated is the bottom-up and top-down attentional processing. The neuropsychophysiological components obtained with this task with greater clinical utility are P3a and P3b, allowing to identify those people with alterations of the attentional functioning as a consequence of a previous vulnerability. In the case of the Go-NoGo paradigm, the cognitive processes evaluated are both the inhibitory capacity and the ability to identify the need to inhibit. In this case, the neuropsychophysiological components obtained with greater clinical utility are N2 and P3, allowing the assessment of this capacity with greater sensitivity than behavioral data.

## Evidence in Other Neuropsychophysiological Measures

Research lines regarding the search for neuropsychophysiological markers of neurocognitive alterations of alcohol dependence are rising as the possibilities for signal analyses increase. This allows us to evaluate EEG signal obtained with more complex paradigms from the methodological and cognitive point of view. By employing different types of analysis of the brain electrical activity, such as frequency analysis methods (102), event-related oscillations (103, 104) and functional connectivity analysis (102).

### Frequency-Based Analysis

In addition to electrical activity related to events, brain oscillations analysis has been used across the literature. Most frequency bands have been evaluated during resting-state recordings in alcohol dependence, although with more diffuse results than those found in ERP studies. As an example, results in resting delta (0, 1–4 Hz) are considered inconclusive (15). With respect to theta band (4–8 Hz), various outcomes have been found (15, 103), but it is fundamentally evidenced as an increase in tonic theta in frontal, central, and parietal areas (105), with greater values for those patients that experience relapse (106). Other studies, however, find a decrease of theta power related to greater cortical damage (107). Changes in theta could be indicating the cortical imbalance in the excitation-inhibition homeostasis (15, 105). Moreover, theta has been related to inhibitory and motor responses (108).

With respect to alpha band (8–12 Hz), that predominates through the resting-state, it seems to be reduced in alcohol-dependent individuals (15, 109) and it has been associated with cortical activations, alert mechanisms, and active inhibition (110, 111), possibly indicating the activation of compensation mechanisms.

For beta band (14–30 Hz), the most frequent result indicates that patients have a greater beta power in fronto-central areas, similar to their offspring, where this increase is produced prior to developing an alcohol use problem (112). There is even evidence of a greater desynchronization of beta in patients that relapse comparing to those that remain abstinent. Porjesz and colleagues support the theory that a greater beta power at frontal sources reflects the disbalance between excitatory and inhibitory neurons, that could underlie to AUDs vulnerability (112–114). In this manner, while findings in beta have been considered as a trait marker, outcomes in theta have been thought as a state marker.

### Event-Related Oscillations

Evoked response oscillations analysis allows us to know in a detailed manner characteristic of brain rhythms during a task performance and their alterations. Within the study of the cognitive function, research groups have mainly found changes in theta band in several behavioral paradigms (34, 115–117).

In relation to incentive salience and motivational-related tasks, alcohol-dependent individuals show a decreased theta power during reward processing and weaker activity in prefrontal sources during the loss condition (34), the latter indicating difficulties in the risk evaluation process. Although a few number of studies are available regarding theta oscillations in substance-cue or reward processing, theta power is found altered in other ERP paradigms, namely visual oddball or conflict tasks in alcohol-dependent individuals (13, 118–120) and their offspring (103). Theta rhythm is thought to reflect frontal activity, implicated in attentional and monitoring processes (121) and it could be implicated in several cognitive operations, such as attentional control, inhibitory processes, and conflict responses.

Theta frequency band has been found to be modulated by stimuli valence. Aftanas et al. (122) measure theta synchronization and desynchronization while healthy subjects are exposed to stimuli with different emotional content, and find a time-locked synchronization theta at anterior and posteriors sites, at 200 to 500 ms post stimuli presentation. This frequency band is useful as a neuropsychophysiological marker of emotional processing. In particular, theta power at frontal areas during the processing of affective information can be useful to assess emotional regulation or control. While theta at occipital sites can be used as a measurement of early emotional assessment. Regarding alcohol effects on theta band in emotional tasks, binge drinkers seem to have an attenuated theta power in an affective appraisal task, during both early appraisal and later integrative processes (115). This lower theta responsitivity to emotions can suggest that binge drinkers already present some of the characteristic anhedonia of AUD patients.

In relation to the executive control dimension, for example, Kamarajan et al. (118) studied the evoked power of brain signals in alcoholics during the performance of a Go-NoGo task. The results showed that alcoholics had lower power in the delta and theta frequency bands compared to the control group in the NoGo condition (118). In this same line, Pandey et al. (117) found that alcoholics had lower power in the delta, theta, and alpha frequency bands compared to the control group, although behavioral differences were found only in the Go condition. Both studies reflect the presence of a neurocognitive deficit in both the execution and suppression of the motor response (117), where, in addition, Kamarajan et al. (118) suggest that oscillatory correlates during cognitive processing may be used as endophenotypic markers in alcoholism. Theta has already been suggested as indicatory of attentional and executive processes (121), hence, these results might indicate a role for this oscillatory activity in detecting and characterizing alterations of these processes during the course of AUDs.

In summary, we could think of brain oscillatory rhythms as possible markers for cognitive processes, such as attentional allocation and affective processing, as well as behavioral monitoring and risk-taking, and executive inhibitory processes, deeply involved in the dependence course and vulnerability toward maladaptive behaviors. In particular, theta and beta rhythms at rest and during cognitive operations could be of interest in the search for clinical or stable biomarkers

### Functional Connectivity of the Brain

Nowadays research focuses on more detailed and global characterization of brain functioning, functional connectivity (FC) and graph theory-based analysis bringing information upon the wiring and effective communication of the brain during several cognitive and emotional processes. In this way, fMRI studies find connectivity changes in the brain affected by chronic alcohol consumption, with an alteration of white matter tracts (123), and changes in attentional and salience networks, as well as reward-related and executive ones (124–126). Coherence analysis or phase-synchrony in several frequency bands between pair of brain regions are frequent ways of studying FC in neuropsychophysiological measures (127). There are several outcomes related to alcohol effects in the brain communication in resting-state. In this way, some studies show a reduced connectivity in theta and alpha, as well as beta band (109, 128). Others show an increase in theta (112, 129–131) and interhemispheric alpha and beta (132). Despite the quite diverse outcomes, there seems to be an agreement upon their role in the brain, and some of them have even been proposed as possible markers of alcohol effects in the brain or as vulnerability markers, such as changes in theta connectivity (131). In this manner, alterations in FC of theta might indicate changes in the inhibitory neuronal system, related to GABAergic and cholinergic neurotransmission (112), as well as emotional and motivational processing (133), whereas beta and alpha FC might reflect supervision and coordination processes involved in brain activation and deactivation systems (134).

Although reduced, there are some studies carried out in active states in EEG regarding alcohol or other substances’ effects on functional connectivity study of the brain. In incentive salience, an fMRI study was carried out in a cue-exposure paradigm, as well as a resting-state EEG recording of alcohol-dependent individuals (130). Results showed fMRI changes in FC in frontal and limbic regions, highly implicated in motivated attention toward alcohol cues, as well as an increased connectivity in theta band in resting EEG in similar regions. Theta is considered to reflect emotional and motivational processes, and this result might underlie to the alterations present in the reward evaluation system in alcohol dependence. The authors of the mentioned study think that theta hyperconnectivity might have a relationship with craving, even hypothesizing the existence of a “central craving network”, where different regions are in charge of appetitive and motivational aspects involved in incentive salience (130). Moreover, this idea is supported by a study with smokers, where theta coherence is increased toward nicotine cues in frontal and parieto-occipital sites, and it also predicts changes in craving (135). Alcohol-related cues could have a similar effect in brain connectivity, in fact, one of our studies (136) showed a relationship between resting-state beta connectivity and the approximation index toward aversive contexts related to the substance in a modified Alcohol Approach-Avoidance task (AAT). Specifically, a higher beta connectivity was related to a greater avoidance of aversive alcohol-related contexts, possibly indicating beta synchronization role in motivational and salience circuits.

Regarding reward-related processing, such as monetary tasks, no studies using EEG or MEG measures were found in FC in alcohol dependence. However, a study carried out in healthy population indicates changes in FC (133), reflected by increases in theta synchronization between frontal region and mostly parietal areas during the loss condition and greater theta FC within posterior regions during the gain condition. Moreover, this study is carried out in co-twins and they find a genetic heritability of fronto-parietal connectivity in theta during the loss condition, indicating that reward evaluation of negative outcomes could be genetically transmitted. Taking this into consideration, it would be interesting to evaluate theta connectivity in incentive or reward tasks and find out its possible role as a vulnerability marker for alterations in reward evaluation and its relationship with maladaptive behavior.

Neuropsychophysiological connectivity measures could also be applied to the assessment of negative emotionality. In healthy population, increased long-range connections between frontal parietal and temporal areas in beta and gamma bands are related to negative and positive emotional information processing (137). In this way, fast rhythms communication through the brain could play a role in emotional processing and could be of use in the research of neural markers of alterations in AUD.

In executive functioning, FC analysis can have a special relevance in the knowledge of electrical brain functioning in resting state and its relation to different executive aspects (e.g., impulsivity). Herrera-Diaz et al. found that alcohol-dependent individuals present, together with an increased beta power, a reduced FC at fronto-central and occipito-parietal regions in alpha and beta bands, comparing to healthy controls. What is more, resting FC in alpha band between anterior and central regions seems to have an inverse relationship with BIS-11 non-planned impulsivity scores (102). Authors propose that results reflect the existence of alterations in the brain’s electrical signal at rest in alcohol-dependent individuals, indicating a possible association to psychopathological characteristics of the addictive behavior (102).

### Brain Graph Characteristics

Research in brain connectivity and dynamics has been increasingly developed during the last years, revealing the importance of neural integration and segregation of communication between brain areas, as well as communication patterns, hubs, and efficiency of the information flow. These particular measures can be useful in different pathologies where functional brain alterations are evidenced (138, 139), as in addictions (140, 141). These measures rely on graph theory, a mathematical model used in several fields of science, particularly useful in neuroscience, more specifically in brain functional and effective connectivity. It comprehends brain functioning as a network, composed by vertices or nodes, e.g., brain areas or channels and edges, e.g., connections (measured by statistical dependence) between pairs of areas (142). The functioning or communication between these nodes is characterized in terms of several elements, such a distance or path length between two areas, the number of connections received by a node (degree) or the number of connections forming a triangle around a node (clustering coefficient) (143). Moreover, the efficacy of this flow of communication depends on the distance between nodes, being inversely related to this index. These characteristics can be calculated at both local (segregation measures) and global (integration measures) levels (144). Segregation refers to the functioning of specialized areas and local networks in the brain, and it can be measured through parameters such as degree, local clustering or local efficiency. Whereas integration involves the coordination of neural populations giving rise to cognitive states, and it is generally measured by indexes such as the average shortest distance (characteristic path length) between nodes, global efficiency and global clustering level. These two principles create diverse and complex patterns of communication in the brain, allowing a balance between flow efficiency and costs, at both resting and during active states. In this way, brain connectivity tends toward a balance in energetic cost and to a maximization of the network (145). This is known as the “small-worldness” of a network and it implies an optimal functioning of local and global communication, characterized by a high global efficiency and clustering level, as well as a short characteristic path length (146).

So, how can these brain graph measures help us in the search for neuroscience-based clinical tools in alcohol dependence? Small-world attributes and characterization of neuropsychophysiological activity patterns can bring answers upon the mapping of functional connections in the altered networks in alcohol dependence. Information processing can be put together with a precise characterization and mapping of the communication flow through the brain, by detecting key nodes or hubs of connections, as well as possible changes in the network organization (147). In other words, we could detect local and global network deficiencies as well as possible compensation mechanisms, with extended communication to other areas or networks. In fact, the available literature upon this theme is still short, but it points toward this type of results. For example, in a fMRI study, individuals with policonsumption show a lower efficiency and a reduced small-worldness of the brain network (140), reflecting a loss of interregional communication in the brain. In alcohol dependence, a smaller global efficiency and clustering level have been related to a greater consumption severity (148), indicating their possible role in identifying neural markers for clinical severity. There is also data in EEG studies, low doses of alcohol in social consumers producing higher global efficiency and an increased density in resting alpha, as well as a short characteristic path (12). Similar observations were made in alcohol-dependent individuals during a working memory task (149), where smaller characteristic paths, reduced clustering, and an increased global efficiency were found in low beta band. These results might be indicating an altered functioning of network efficiency under the effects of alcohol, as well as a compensating mechanism in response to task demands, in order to carry out cognitive operations. Hence, graph-based measures of brain connectivity using neuropsychophysiological measures could help explain in detail neural communication alterations that happen through the course of dependence.

## Discussion

Brain electrical activity has been used with some degree of evidence to evaluate several of the cognitive processes that are contemplated in the dimensions of ANA. These cognitive components may describe alcohol dependence as an active process, in which event-related potentials or oscillations serve as a measure of the progression of the disorder. For instance, in relation to stimuli relevance evaluation, a greater alcohol salience can be observed in early visual ERP components, reflecting motivational processes. A common finding is a greater amplitude and sometimes latency of P1, N1, or P2 components, reflecting a preferential attention toward alcohol and related cues. This preferential attention can affect other cognitive processes, such as the inhibitory capacity, reflected by greater N2 amplitudes when alcohol-dependent individuals carry out an inhibitory process in presence of alcohol cues, dampening behavioral control. Moreover, incentive salience is also evidenced through higher P3 amplitudes in frontal regions and a reduced power of theta band, during loss and gain conditions in decision-making tasks. This supports the behavioral results that indicate difficulties within the proper evaluation of behavioral alternatives in AUDs. In the clinical context, patients with more altered values in this dimension put alcohol at the center of their thinking, without attending to other things in the environment. As a consequence, the risk of relapse in situations where alcohol is present is greater.

Patients are not always aware of this attentional bias, so a good therapeutic proposal would be the training in attentional control and avoidance of risky situations at least at the beginning of treatment. As treatment progresses, work through associative learning could be targeted to establish new contingencies, new stimulus-response-reinforcement relationships. At this point the patient could address a re-evaluation of the way he makes decisions, to value the new reinforcements and train the delay of the reward (see **Table 1**).

**Table 1:** Behavioral and ERP/ERO paradigms within ANA framework (Incentive salience).

ANA dimension	Paradigm	Psychophysiogical variable	Cognitive processes assessed	Population	Treatment target	Vulnerability	Severity	Outcome	Course
Incentive salience	*Cue reactivity task*	N1: higher amplitude	Preferential attentional processing of substance cues	Alcohol dependence	Attentional bias/Associative learning	?	+++	?	?
		P1: higher latency	Faster early attentional processing of alcohol cues	Social drinkers		?	+++	?	?
	*Dot-probe attentional bias task*	P1: higher amplitude and latency	Preferential early attention orientation	Individuals at risk (low alcohol sensitivity)	Attentional bias/Associative learning	+	?	?	?
		Larger negativity between 220 and 280 ms	Difficulties reorienting attention away from alcohol cues	Individuals at risk (low alcohol sensitivity)		+	?	?	?
	*Monetary incentive delay task*	P300: decreased amplitude	Task demands processing and context updating Attentional resource allocation Decision making	Alcohol dependence and offspring	Cognitive evaluation process and decision making	+	+	+	++
		Theta: reduced power	Reward processing	Alcohol dependence		+	+	+	?

This table contains a brief summary of main ERP components, ERO activity and their alterations related to alcohol effects, within ANA’s domain Incentive salience. It also makes a small mention to main cognitive functions that can be targeted inside psychotherapeutic programs. Shadowed columns refer to the evidence gathered by each neuropsychophysiological marker in the evaluation of cognitive and motivational processes of alcohol dependence in four steps: vulnerability toward alcohol use disorders, dependence severity, outcome and course. “<+++> indicates consistent outcomes in the literature, <++> indicates the presence of some studies, although inconclusive, <+> implies an incipient line of study, with preliminary results and finally, <?> indicates a future line of work. Potential clinical use and treatment targets.

Although the evidence in negative emotionality is scarcer and at the time being is still far from being used as a possible endophenotype for AUDs, it could give rise to promising results. In this manner, we could consider neuropsychophysiological activity under affective processes as markers for processes we know as altered in alcohol dependence. To this point, the bibliography considers the use of P1, N1, P2 and LPP components as evidence for the deficient processing of affective content. In addition, there is a promising line in which there is evidence for theta frequency band. Theta seems to be less modulated by affective content in binge drinkers (115), possibly indicating a reduced sensitivity to negative and positive affect. In this way, those people with less recognition of the emotional content, and less adjustment to it, evidenced by the electrical activity, will have greater difficulties in interpersonal relationships (59). Difficulty in recognizing one’s own emotions and those of others can lead on the one hand to interpersonal conflicts, which are considered to be one of the main risk factors for relapses (150, 151). There is also evidence of how the presence of negative affect can impair performance in other areas. For example escape drinkers, that is, individuals motivated to drink in order to avoid negative emotions, show greater N2 amplitudes, implicated in more controlled attentional operations, indicating a greater attentional bias toward alcohol cues (39). Hence, the presence of negative affect seems to lead to greater attention being paid to alcohol-related stimuli. Moreover, it could lead to the use of alcohol as the most effective mechanism for emotional self-regulation. Thus, a treatment goal for this patient profile should first impact on the identification of both negative and positive emotions and the acceptance that negative emotions are adaptive. Once the patient is aware of his or her emotional state, the therapeutic objective could be emotional regulation and adaptive coping (see **Table 2**).

**Table 2:** Behavioral and ERP/ERO paradigms within ANA framework (Negative emotionality).

ANA dimension	Paradigm	Psychophysiogical variable	Cognitive processes assessed	Population	Treatment target	Vulnerability	Severity	Outcome	Course
Negative emotionality	*Affective images processing*	LPP: decreased amplitude to negative images.	Emotional cues classification and processing	Acute alcohol intake Binge drinkers	Affect regulation and coping	+	?	?	?
	*Appraisal*	Theta: decreased power	Emotional processing	Binge drinkers	Re-appraisal	+	?	?	?
	*Emotional Face expressions*	P100, N100, and N170: decreased amplitude.	Recognizing emotional face expressions	Alcohol dependence	Conscious affect processing	?	?	?	?

This table contains a brief summary of main ERP components, ERO activity and their alterations related to alcohol effects, within ANA’s domain negative emotionality. It also makes a small mention to main cognitive functions that can be targeted inside psychotherapeutic programs. Shadowed columns refer to the evidence gathered by each neuropsychophysiological marker in the evaluation of cognitive and motivational processes of alcohol dependence in four steps: vulnerability toward alcohol use disorders, dependence severity, outcome and course. “<+++> indicates consistent outcomes in the literature, <++> indicates the presence of some studies, although inconclusive, <+> implies an incipient line of study, with preliminary results and finally, <?> indicates a future line of work. Potential clinical use and treatment targets.

Finally, the most conclusive results are in the P3 component (see **Table 3**), where both alcohol-dependent patients and their offspring show alterations. Which represents a clear example of a possible endophenotype. This component reflects controlled processes of conscious attention, context updating processing and executive attention (15, 152), that are clearly comprised inside the executive dimension of ANA. In this dimension, there is also evidence in N2, and the delta, theta and alpha frequency bands, reflecting alterations in inhibitory control. The patient with alterations in this cognitive domain will be especially vulnerable to relapses, as it will be difficult to stop automatic behaviors in benefit of more controlled processes, such as rejecting the tendency to consume alcohol one they are inside a context of risk (153).

**Table 3:** Behavioral and ERP/ERO paradigms within ANA framework (Executive dimension).

ANA dimension	Paradigm	Psychophysiogical variable	Cognitive processes assessed	Population	Treatment target	Vulnerability	Severity	Outcome	Course
Executive Dimension	*Oddball Paradigm*	P3a and P3b: reduced amplitude. P3: reduced Delta, theta: decreased power	Attentional resource allocation	Alcohol dependence Offspring Offspring	Attentional control	+++	+++	+++	+
	*Go-NoGo*	N2: reduced amplitude	Inhibition of the motor response	Alcohol dependence	Executive behavior: Identification of high-risk situations. Generation of alternative responses	+	++	++	+
		P300: reduced amplitude	Inhibition of the motor response	Alcohol dependence and offspring		++	+++	+++	?
		Delta	Attention and task demand			+	?	?	?
		Theta	Attention and inhibition	Alcohol dependence		++	?	?	?
		Alpha	Inhibition			+	?	?	?

This table contains a brief summary of main ERP components, ERO activity and their alterations related to alcohol effects, within ANA’s domain executive function. It also makes a small mention to main cognitive functions that can be targeted inside psychotherapeutic programs. Shadowed columns refer to the evidence gathered by each neuropsychophysiological marker in the evaluation of cognitive and motivational processes of alcohol dependence in four steps: vulnerability toward alcohol use disorders, dependence severity, outcome and course. “<+++> indicates consistent outcomes in the literature, <++> indicates the presence of some studies, although inconclusive, <+> implies an incipient line of study, with preliminary results and finally, <?> indicates a future line of work. Potential clinical use and treatment targets.

Taking into account what has been reviewed so far, the combination of information from neuropsychology, clinical psychology, and neuropsychophysiological markers could lead to differentiated clinical profiles, based not on the final behavior of pathological consumption, but on the underlying cognitive processes. The objective for a future line of research would be to adjust the treatment to the deficient processes and to the preserved ones. In addition, the development of this line of work, perhaps can lead us to explain the clinical heterogeneity that we find in daily practice. Thus, as an example for illustrative purpose, a patient with an A profile (see **Table 4**), who manifests both moderate levels of incentive salience and negative emotionality, and a mild affectation of the executive component, could find himself in situations of risk when exposed to the context of consumption, or in moments of emotional conflict. But he would only relapse when his executive control capacity would fail. Possibly his relapse would be characterized by a consumption not necessarily intense and with a determined duration until he could recover the executive control. However, the patient with a B profile (see **Table 4**), under the same risk conditions, but with severe impairment of the executive function, would hypothetically relapse more easily, leading to a relapse of a greater intensity and prolonged in time.

**Table 4:**
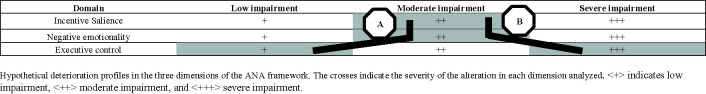
Examples of cognitive profiles in AUD based on cognitive and ERP/ERO evidence.

This future line of work would help design long-term treatment strategies, which considers the starting point, but also the evolution of these cognitive processes within the process of change caused by treatment and abstinence, and therefore it would be individualized (1, 2). Thus, within the framework of techniques that already have proven efficacy, such as contingency management, relapse prevention, motivational interviewing, or pharmacotherapy, the A-profile patient could follow a standardized treatment as a basis, but with an emphasis on recognizing risk situations and formulating alternative plans for consumption. While the B-profile patient, in addition to following the standardized treatment, could reinforce the therapy, by carrying out in the initial moments of the treatment a good management of contingencies, avoiding the situations related to alcohol, and then working explicitly with techniques of compensation or substitution of the inhibitory control.

This line of work has results on specific components such as the attention bias, approximation trends toward alcohol, and the underlying neuropsychophysiological measures such as alpha power or P3 amplitude, which seem to change after treatment (136, 154, 155), being good markers of interindividual change. Therefore, there could be evidence of greater homogeneity when used to assess the clinical course of patients. Since they show a great sensitivity to change along the treatment process (155, 156). Possibly because they reflect the integrity of some cognitive components that we could consider as intermediate endophenotypes, or as “stepping stones” to the disorder, but also toward the clinical recovery (8). This is a very promising line of work.

Notwithstanding, given the variability of the revised measures, the applicability of this proposal is still developing, in order to be accessible for the different health care professionals. Patrick et al. revise the specific needs for implementing these types of measures in our daily clinical practice (10), highlighting several of them. The first one has to do with the need for technological resources: although EEG equipment is habitual in hospital units, they are not always prepared for a complex event-related activity measurement. Additionally, this evaluation supposes an extensive methodological effort, this is probably the greatest difficulty encountered for results replicability and their employment. The second important need has to do with the generalization of the results and their consistency. A great diversity of cognitive and behavioral paradigms is available for cognitive and EEG evaluation, with different types of stimuli, diverse presentation times and even required responses from individuals. This could explain part of the variability found in research, thus a need for studies that verify validity and reliability of the proposed measures is becoming relevant. In this way, standardization and validation processes of behavioral event-related potentials paradigms would be enabled, similar to those carried out to develop neuropsychological assessment (10).

Despite the variability, most of the groups working in this line of research agree in highlighting several advantages of including electrophysiological variables as objective measures of the cognitive process, since their alteration may be present in patients whether or not the final behavior is successful, reflecting the lack of efficiency of the process (10). In this way, the results of neuropsychological and behavioral evaluation sometimes do not identify the high effort that the patient makes to reach successful behavior. Therefore, they do not identify either the lack of resources to cope with more demanding situations, since resources are destined to frequent events. Leaving the patient unable to adjust to more difficult or novel situations. That may make more difficult reinsertion into the different life spheres for the patient with alcohol dependence and in the final recovery achievement (11, 157). Thus, the employment of these types of measures brings a certain type of information that would otherwise pass unnoticed in the clinical context and that could be relevant in order to obtain a more objective profile of the three dimensions proposed by ANA. In the end, this would help into the development of adequate individualized treatment designs (1).

In conclusion, the neuropsychophysiological evaluation through event-related potentials is already set for being employed in the clinical context, although a more extensive diffusion and a process of standardization of paradigms are still necessary, so that professionals can acknowledge its use and for a greater applicability inside personalized interventional programs.

Future lines of work can focus on the use of novel measures of brain communication in AUD characterization. Functional and effective connectivity analyses of the brain can bring light upon the synchronization and coordination between regions and networks that take place during cognitive processing. Moreover, brain graph measures can help us observe specific brain areas that may be acting as hubs of communication, as well as network efficiency at local and global levels in several cognitive functions. In this way, we could observe specific network functioning and interacting from preattentional processes to more controlled and complex mental operations. This is interesting for AUD alterations, since several cognitive domains present deficiencies, considering the early salience of alcohol cues and problems in the affective processing and the executive function. To this date, functional connectivity studies in psychiatric conditions are mostly exploratory and have the purpose of characterizing the neural functioning of the brain. In AUD, functional connectivity studies using EEG have found alterations in brain synchronization in several frequency bands (alpha, beta or gamma) (132, 149, 158) and they seem to present a reduced efficiency of communication, although results are somehow scarce and diverse. Nonetheless, prospective studies should be able to offer clearer hypotheses on brain alterations in psychiatric patients, such as AUD, and to help find out if they persist in time or are modulated by different clinical factors.

## Author Contributions

RJ-B, JP-M, and GR performed the *Introduction* and the *Discussion* sections. AS performed the *Incentive Salience* section. ID-C performed the *Negative Emotionality* section. AM-M performed the *Executive Dimension*. RJ-B, GR, AS, ID-C, and AM-M contributed to the performance of the *Other Psychophysiological Evidences, Brain Functional Connectivity*, and *Network Characteristics* sections. FN, MG-G, and JM have collaborated in the manuscript planning and reviewing, as well as the writing supervision in terms of relevance for the reader. JM contributed by selecting the most relevant information for the aim of the publication. FN and MG-G have made a revision of the English version of the manuscript.

## Conflict of Interest

The authors declare that the research was conducted in the absence of any commercial or financial relationships that could be construed as a potential conflict of interest.
